# Analysis of Association between Vitamin D Deficiency and Insulin Resistance

**DOI:** 10.3390/nu11040794

**Published:** 2019-04-06

**Authors:** Izabela Szymczak-Pajor, Agnieszka Śliwińska

**Affiliations:** Department of Nucleic Acid Biochemistry, Medical University of Lodz, 251 Pomorska, 92-213 Lodz, Poland; izabela.szymczak@umed.lodz.pl

**Keywords:** insulin resistance, insulin-responsive tissues, vitamin D, pancreatic β-cells dysfunction, sub-inflammation, oxidative stress

## Abstract

Recent evidence revealed extra skeleton activity of vitamin D, including prevention from cardiometabolic diseases and cancer development as well as anti-inflammatory properties. It is worth noting that vitamin D deficiency is very common and may be associated with the pathogenesis of insulin-resistance-related diseases, including obesity and diabetes. This review aims to provide molecular mechanisms showing how vitamin D deficiency may be involved in the insulin resistance formation. The PUBMED database and published reference lists were searched to find studies published between 1980 and 2019. It was identified that molecular action of vitamin D is involved in maintaining the normal resting levels of ROS and Ca^2+^, not only in pancreatic β-cells, but also in insulin responsive tissues. Both genomic and non-genomic action of vitamin D is directed towards insulin signaling. Thereby, vitamin D reduces the extent of pathologies associated with insulin resistance such as oxidative stress and inflammation. More recently, it was also shown that vitamin D prevents epigenetic alterations associated with insulin resistance and diabetes. In conclusion, vitamin D deficiency is one of the factors accelerating insulin resistance formation. The results of basic and clinical research support beneficial action of vitamin D in the reduction of insulin resistance and related pathologies.

## 1. Introduction

Type 2 diabetes (T2DM) is increasingly common and alarming both national and worldwide [1]. The World Health Organization reported that estimated 90% of all cases of diabetes constitutes T2DM and approximately 15 million people globally suffer from T2DM. Moreover, this number might be doubled by 2025 [2]. The following disturbances: systemic inflammation, defects in insulin signaling pathway, and pancreatic β-cells dysfunction, are engaged in both insulin resistance and T2DM development [1].

Currently, vitamin D deficiency seems to be frequent and related to pathogenesis of numerous diseases, including metabolic abnormalities [1]. The association between vitamin D deficiency and insulin resistance has been also proposed [2]. Numerous clinical studies showed that vitamin D supplementation reduces the level of metabolic parameters such as total cholesterol (TC), low-density lipoprotein (LDL), triglyceride (TG), glycated hemoglobin (HbA1c), as well as decreases insulin resistance indicator—HOMA-IR—in T2DM patients [3,4,5,6,7]. However, it is not fully recognized how vitamin D may reduce the risk of metabolic disorders development. Recently, vitamin D receptor (VDR) and vitamin D-metabolizing enzymes were detected in various cell types, including pancreatic β-cells and insulin-responsive cells such as adipocytes. Adipose tissue is a major site of vitamin D storage and an important source of adipokines and cytokines participating in the formation of systemic inflammation [8]. It is well known that obesity, especially visceral, is one of the major risk factors for T2DM. It has been also suggested that the potential link between diabetes and obesity is vitamin D deficiency coexisting with obesity [9]. Evidence suggests that vitamin D seems to be a regulator of numerous sequential events that are responsible for enabling the pancreatic β-cells to secrete insulin, and thereby to control of blood glucose level.

**Figure 1:**
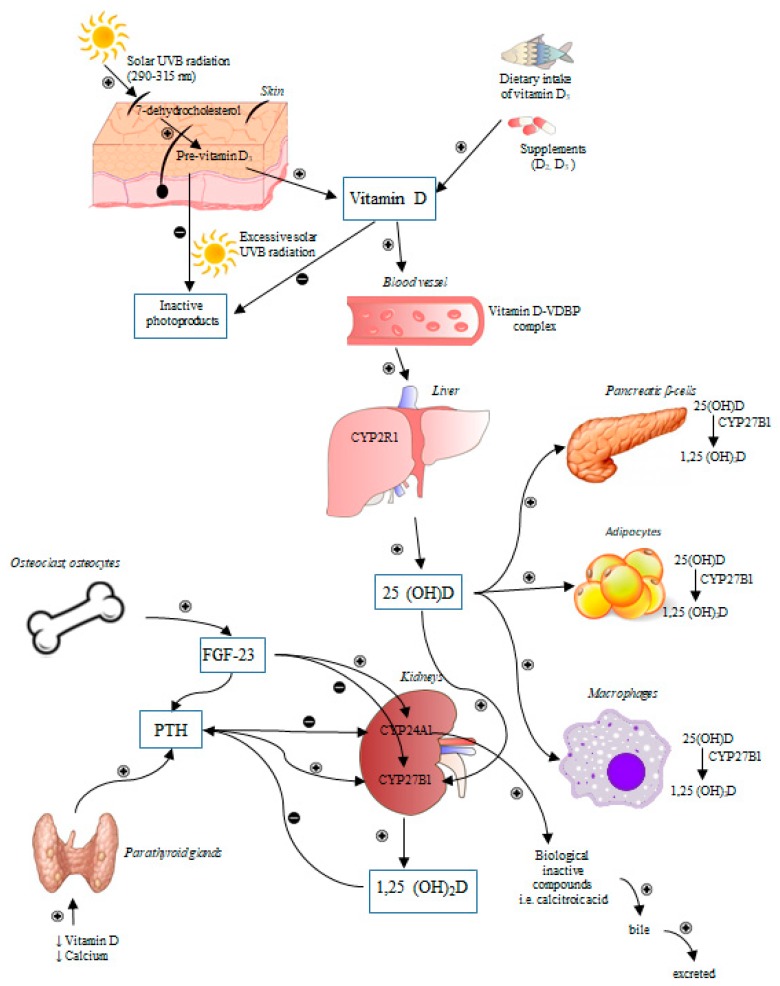
The regulation of synthesis and metabolism of vitamin D. Under ultraviolet radiation (UVB, 290–315 nm) action, 7-dehydrocholesterol in converted into previtamin D_3_ in the skin. In turn, previtamin D_3_ is immediately transformed into vitamin D_3_ as a result of heat-dependent process [10]. During excessive exposure to sun, previtamin D_3_ and vitamin D_3_ are broken down into inactive photoproducts to prevent vitamin D_3_ intoxication [11]. Both vitamin D_2_ and vitamin D_3_ derived from synthesis in the skin and a diet may be transported by vitamin D binding protein (VDBP) with the bloodstream or may be stored in adipocytes and then released to the circulation. The next step of vitamin D metabolism comprises two consecutive enzymatic hydroxylation reactions leading to vitamin D activation. The first step of vitamin D activation is the formation of 25(OH)D in the liver by vitamin D-25-hydroxylase, a cytochrome P450 enzyme, (mainly CYP2R1) [12]. The 1,25(OH)_2_D (calcitriol, the bioactive metabolite of vitamin D) forms as a result of 25(OH)D hydroxylation being performed by 25(OH)D-1α-hydroxylase (CYP27B1). This enzyme is present not only in the tubules of kidney, but also in numerous cells including macrophages, adipocytes, and the pancreatic β-cells [13,14,15,16]. The 1,25(OH)_2_D_3_ is able to induce its own degradation via the stimulation of 25(OH)D-24-hydroxylase (CYP24A1). CYP24A1 is an enzyme responsible for the degradation of both calcitriol and its precursor 25(OH)D to biological inactive metabolites, i.e., calcitroic acid excreted with the bile [11]. A low level of vitamin D and calcium stimulates parathyroid gland for the release of parathyroid hormone (PTH) and induction of CYP27B1 synthesis, resulting in elevated calcitriol activation [17]. The 1,25(OH)_2_D_3_ may reduce its own synthesis via negative feedback loop and decreases both synthesis and secretion of PTH. PTH is also capable of inhibition of CYP24A1 [18] and induction of skeletal fibroblast growth factor 23 (FGF-23) synthesis [19]. FGF-23 regulates the vitamin D homeostasis via inhibiting renal expression of CYP27B1 and stimulating expression of CYP24A1 which resulting in the reduction of calcitriol level in the serum [11]. 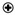
—stimulation, 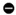
—inhibition.

The term of vitamin D refers to its two major forms: vitamin D_2_ (ergocalciferol) and vitamin D_3_ (cholecalciferol). Humans acquire vitamin D from the exposure to sunlight, ingested food and dietary supplements. The Figure 1 presents mechanisms responsible for the synthesis and metabolism of vitamin D [10].

Vitamin D exerts an effect on gene transcription via genomic and non-genomic mechanisms of action. Genomic mechanism is mediated via VDR, that belongs to the family of nuclear receptors and acts as a ligand-activated transcription factor. The active form of vitamin D, 1,25(OH)_2_D_3,_ binds to VDR, which in turn forms heterodimer with the retinoid receptor (RXR). Then, the complex of 1,25(OH)_2_D_3_–VDR–RXR is translocated to the nucleus, where it binds to vitamin D-responsive elements (VDRE) in the promoter region of vitamin D-responsive genes. Interaction between 1,25(OH)_2_D_3_–VDR–RXR and VDRE results in the recruitment of diverse enzymatic coregulatory complexes that are responsible for the chromatin remodeling, facilitating of the epigenetic modification of histones as well as local RNA polymerase II recruitment. These changes positively or negatively regulate positively or negatively the expression of target genes, including these responsible for proliferation and differentiation of cells, immunomodulatory activity and angiogenesis [20,21,22].

Non-genomic action of vitamin D is manifested as the activation of numerous signaling molecules (i.e., phosphatidylinositol-3 kinase, phospholipase C (PLC), Ca^2+^-calmodulin kinase II (CaMPKII), protein kinase A (PKA), mitogen-activated protein kinases (MAPK)s, src, protein kinase C (PKC)). Targets of these kinases are transcription factors (i.e., SP1, SP3, and RXR) that in turn interact with VDRE on the promoter of vitamin D-responsive genes. Vitamin D is also engaged in the production of second messengers (i.e., cyclic AMP, Ca^2+^, fatty acids, and 3-phosphoinositides). The range of activated signaling molecules is associated with the type of cell and its status of maturation [23].

The indicator of vitamin D level is the concentration of its circulating metabolite, namely 25(OH)D, whose half-life time is 10–19 days [13]. The level of 25(OH)D reflects the level of vitamin D that comes from the synthesis in the skin and dietary intake. Several institutions and societies, including American Society of Endocrinology or Institute of Medicine, have developed guidelines regarding vitamin D levels which determine the status of vitamin D deficiency, insufficiency, and sufficiency. Classification of established diagnostic vitamin D cut-offs based on 25(OH)D concentration according to Alshahrani et al. [24] is presented in Table 1.

**Figure 2:**
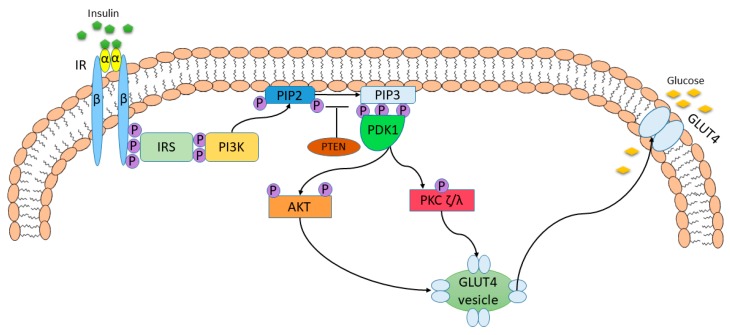
The insulin signaling pathway under physiological condition. Insulin action is initiated via its binding to insulin receptor (IR). The activation of IR contributes to the dimerization of the receptor and generation of the heterotetrameric form. Autophosphorylation of IR leads to the formation of numerous phosphotyrosine residues which are potential docking sites for the component of other signaling pathways [29]. The recruitment and phosphorylation of numerous substrate proteins, including insulin-receptor substrate (IRS) proteins, are allowed via multiple phosphotyrosines [30]. Phosphorylated IRSs activate and translocate phosphatidylinositol-3-kinase (PI3K) to the plasma membrane, and PI3K phosphorylates phosphatidylinositol 4,5-biphosphate (PIP2) to phosphatidylinositol-3,4,5-biphosphate (PIP3)—a key lipid signaling molecule. The level of PIP3 is under control of phosphatase and tensin homolog (PTEN) and SH2-containing inositol 5′-phosphatase-2 (SHIP2) that perform PIP3 dephosphorylation [28]. Insulin-mediated elevation of PIP3 level induces serine threonine kinase PDK1 (phosphoinositide-dependent protein kinase-1), thus leading to the phosphorylation and activation of protein kinase C (PKC ζ/λ) and protein kinase B (PKB also known as AKT). One of their actions is the translocation of glucose transporter 4 (GLUT4) to cell membrane and, in consequence, the elevation of glucose uptake [31]. AKT also stimulates synthesis of protein, glycogenesis, and lipogenesis, but represses lipolysis, glucogenolysis, gluconeogenesis, and proteolysis [28].

Insulin is a major regulator of carbohydrate and lipid metabolism [25]. Increase of glucose in blood induces the secretion of insulin by the pancreatic β-cells [26]. Glucose enters the pancreatic β-cells via the glucose transporter 2 (GLUT2), where is converted into fructose-2,6-P_2_ (F-2,6-P_2_). F-2,6-P_2_ enters the tricarboxylic acid cycle and glycolytic pathway leading to increase of ATP level. Elevation of ATP inhibits the ATP-sensitive K^+^ channel resulting in the depolarization of membrane that causes activation of the L-type voltage-operated channels to generate the localized Ca^2+^ pulses triggering the secretion of insulin. [27] Figure 2 presents the insulin signaling pathway under physiological condition [28].

**Figure 3:**
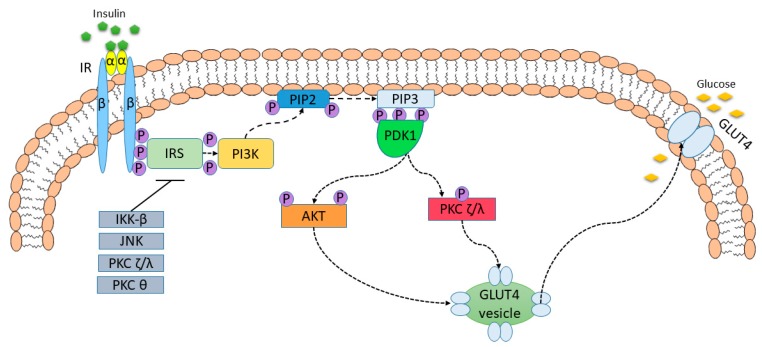
The attenuation of insulin signaling pathway in insulin resistance condition. Numerous protein kinases, i.e., IKK-β, JNK, PKC ζ/λ, PKC-θ, contribute to the phosphorylation of IRS that in turn attenuate insulin signaling. This state is presented in insulin resistance. 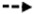
—attenuation.

The disturbances in insulin pathway are responsible for the development of insulin resistance. Insulin, by the control of numerous enzymes and kinases during feeding and fasting periods, is a major regulator of energy homeostasis [32]. Thereby, the decline of insulin capability to elevate the uptake of glucose by adipose tissue, liver, and muscle, contributes to the development of insulin resistance [25]. It was found that inhibitory effect of insulin on lipolysis is diminished during decreased insulin sensitivity. As a result, the level of circulating free fatty acids (FFAs) increases. FFAs may be taken up by liver, muscles, and pancreas [33]. Consequently, non-adipose tissue insulin resistance is developed as a result of lipotoxicity. Several molecular pathways have been proposed as playing important roles in this disorder [32]. It was demonstrated that FFAs and related metabolites including ceramides, acyl-CoA, diacyloglycerol via acting on numerous protein kinases, i.e., nuclear factor-κB (NF-κB) kinase-β [IκB kinase-β (IKK-β)], Jun kinase (JNK), PKC ζ/λ, PKC-θ contribute to the phosphorylation of IRS that in turn attenuate insulin signaling [33,34,35,36]. Figure 3 presents the attenuation of insulin signaling pathway in insulin resistance condition.

White adipocytes store fat, if calories intake exceeds the needed amount [28]. However, this prolonged state causes hyperplasia and hypertrophy of adipocytes as well as adipose tissue hypoxia. The result of these undesired disturbances is low grade chronic inflammation (sub-inflammation) that accompanies insulin resistance [37]. In this condition, insulin is not able to further stimulate energy stored in adipocytes. The pancreatic β-cells undergo adaptive changes leading to the production and secretion of large amount of insulin creating state of hyperinsulinemia [33]. Hyperinsulinemia triggers the pancreatic β-cells exhaustion resulting in decline of their mass [38]. Finally, when the reduction of the pancreatic β-cells mass up to 60%, T2DM is diagnosed [28]. Overstimulation of pancreatic β-cells in insulin resistant state contributes to the elevated level of Ca^2+^ and overstimulation of insulin secretion [39,40]. Thus, the excessive Ca^2+^ signaling is involved in death of the pancreatic β-cells [38,41,42,43,44,45].

Growing evidence revealed that insulin resistance is also closely related to obesity and coexisting oxidative stress as well as low grade inflammation [33,46]. Reactive oxygen species (ROS) act as a signaling molecules that activate numerous cellular stress-sensitive pathways, i.e., NF-κB, JNK/SAPK, p38MAPK, and hexosamine, involved in cellular damage and inflammation, both of which are associated with pancreatic β-cells dysfunction, insulin resistance, and diabetices complications [47].

An accumulating amount of data suggests that vitamin D deficiency may be involved in the pathomechanism of metabolic abnormalities leading to hyperglycaemia and obesity [48]. Furthermore, vitamin D might play a crucial role in modifying the risk factors for T2DM [49]. It can be mediated by improvement of the pancreatic β-cells function, insulin sensitivity, and decrease of systemic inflammation [8,50,51]. Therefore, the aim of this paper was to present mechanistic context relating to vitamin D deficiency and insulin resistance formation.

## 2. Materials and Methods

To provide the current findings regarding the molecular role of vitamin D deficiency in the insulin resistance formation, the PUBMED database (http://www.ncbi.nlm.nih.gov/pubmed) and published reference lists were searched to identify pertinent articles published between 1980 and 2019. The combination of the following keywords was used: vitamin D OR vitamin D deficiency AND insulin resistance OR insulin signaling OR insulin sensitivity OR insulin secretion OR pancreatic β-cells dysfunction OR sub-inflammation OR oxidative stress. This narrative review presents the results of in vitro, animal and human in vivo studies, including clinical trials.

## 3. Is Vitamin D Level Related with Insulin Resistance—Results of Observational and Interventional Clinical Trials

The results of numerous observational studies revealed that hypovitaminosis D favors the development of insulin resistance since serum vitamin D levels correlated with the values of metabolic parameters, including BMI, HOMA-IR, TG, HDL, LDL, TC, and HbA1c. Table 2 provides details of selected observational studies regarding the association between vitamin D levels and metabolic parameters. The results of the majority of interventional clinical trials confirmed the beneficial effect of vitamin D supplementation on insulin sensitivity. However, there are studies presenting the lack of any impact of vitamin D on metabolic parameters associated with insulin resistance in T2DM and prediabetic patients as well healthy subjects. Table 3 presents selected interventional clinical trials aimed to determinate effect of vitamin D supplementation on metabolic parameters connected with insulin resistance.

## 4. Molecular Mechanisms of Relationship between Vitamin D Deficiency and Insulin Resistance

### 4.1. Vitamin D Maintenances Pancreatic β-Cells Function

Results of pre-clinical studies have shown that vitamin D seems to be a potential regulator of insulin secretion, Ca^2+^ level, and survival of the pancreatic β-cells. Several studies have demonstrated that vitamin D deficiency contributes to impairment of glucose-mediated secretion of insulin in rat pancreatic β-cells [71,72,73,74,75]. It was also reported that glucose-mediated secretion of insulin seems to be restored via vitamin D supplementation [71,72,76]. The results of some clinical studies [77,78,79], but not all [51,80], have shown that vitamin D supplementation was associated with the improvement of insulin secretion [81].

Both VDR and CYP27B1 are expressed in the pancreatic β-cells. Thus, vitamin D action in the pancreatic β-cells seems to be exerted directly via binding of vitamin D to VDR [81,82]. Mice with the lack of functional VDR presented impaired secretion of insulin after glucose load. It was also associated with decreased synthesis of insulin by the pancreatic β-cells resulting in the reduction in the amount of stored insulin [83]. Calcitriol may directly stimulate secretion of insulin because VDRE was identified in the promoter of insulin gene in the pancreatic β-cells [84]. Interestingly, VDRE induced not only the transcription of insulin gene, but also other numerous genes involved in cytoskeletal organization, intracellular junctions and cellular growth of pancreatic β-cells [85].

Calcium is an essential component for proper undergoing of numerous insulin-mediated intracellular processes in target tissues, i.e., muscle and adipose tissue. Optimal intracellular level of Ca^2+^ is indispensable for proper insulin action. Impaired transduction of insulin signaling, being related to decreased activity of glucose transporter as a result of alterations of intracellular Ca^2+^ in target tissues, may lead to peripheral insulin resistance. The 1,25(OH)_2_D exerts an effect on insulin sensitivity via regulation of extracellular Ca^2+^ concentration and its flux through cell membranes [76]. It has been also observed that vitamin D deficiency contributes to increasing Ca^2+^ concentration that may decrease GLUT-4 activity leading to insulin resistance [86,87].

Vitamin D is engaged in regulation of Ca^2+^ flux in the pancreatic β-cells. 1,25(OH)_2_D_3_ decreases expression of the L-type Ca^2+^ channels leading to alteration in Ca^2+^ signaling. Rapid, non-genomic mechanism of vitamin D action was found to be involved in increasing of cytoplasmic Ca^2+^ level that stimulates exocytosis mechanism of insulin secretion in the pancreatic β-cells. This effect was mediated via activation of two signaling pathways. The first of them includes activation of PKA that phosphorylates different proteins involved in the role of L-type voltage-dependent Ca^2+^ channels related to increase of insulin secretion. The second of these signaling pathways involves activation of IP3 synthesis and PLC synthesis, which contributes to the release of Ca^2+^ from ER and diacyloglycerol (DAG) that in turn activates PKC. The activated PKC is responsible for phosphorylation of the K_ATP_ channels and L-type voltage-dependent Ca^2+^ channels. All of these processes lead to the depolarization of cytoplasmic membrane and opening of L-type and T-type Ca^2+^ channels, elevating intracellular Ca^2+^, which then stimulates secretion of insulin [84]. PKC is also able to mobilize the secretory vesicles which together with elevated Ca^2+^ level promote secretion of insulin [88]. It was also shown that increased Ca^2+^ level results in the insulin secretion via activation of CaMKII. CaMKII is a serine threonine protein kinase localized in insulin secretory vesicles. Its main function is the promotion of phosphorylation of proteins involved in both mobilization and exocytosis of insulin vesicles. It has been also proposed that increased intracellular Ca^2+^ level might induce the expression of insulin gene via cAMP-responsive Element-binding Protein (CREB). CREB is an important transcriptional element involved in the maintenance of efficient insulin gene transcription, glucose sensing, pancreatic β-cells survival, and insulin exocytosis [89]. Moreover, calcitriol also regulates calbidin-D_28k_ expression. Calbidin-D_28k_ is a cytosolic Ca^2+^-binding protein involved in the stimulation of insulin secretion via regulation of intracellular Ca^2+^ level [81,90]. Vitamin D also increases expression of parvalbumin, calbindin D-9k, the sodium/calcium exchanger (NCX), and the plasma membrane Ca^2+^-ATPase 1b and the Ca^2+^ pumps. All of these proteins are responsible for maintaining low resting Ca^2+^ level [91,92,93]. To conclude, vitamin D seems to be a modulator of depolarization-stimulated secretion of insulin via intracellular Ca^2+^ regulation [90].

### 4.2. The Effect of Vitamin D on Insulin Signaling and Sensitivity

Vitamin D is involved not only in the function of pancreatic β-cells, but also in insulin-responsive tissues, including adipose tissue, liver, and skeletal muscle [84]. The results of several studies have shown that vitamin D increases insulin sensitivity that might be mediated via binding of 1,25(OH)_2_D_3_ to VDR [94], induction of IRs expression on target tissues [95], as well as activation of PPAR-δ [96]. Vitamin D is able to stimulate IRs in target insulin-responsive tissues. In insulin-responsive cells, 1,25(OH)_2_D interacts with VDR which in turn binds to RXR. Then, 1,25(OH)_2_D_3_-VDR-RXR complex binds to VDRE in the promoter of human insulin receptor gene. As a result, transcriptional activation of IR gene is enhanced and the number of IRs increase [26]. Elevated expression of IR gene maintains proper insulin signaling pathway [95,97]. Thus, the active metabolite of vitamin D seems to be a stimulator of IR expression, which in turn improves insulin sensitivity [95,97,98]. This data confirm that vitamin D deficiency seems to be engaged in the onset of insulin resistance due to down-expression of IR [50]. Interestingly, the results of vitamin D-mediated stimulation of IR expression in the liver are unambiguous. George et al. have shown that vitamin D supplementation increased liver expression of IRs in streptazotocin-induced diabetic rats [99]. On the other hand, several studies have not demonstrated changes in IRs expression in the liver in mice fed with low-fat diet or high-fat diet [100] as well as in streptozotocin-induced diabetic rats [101].

Interestingly, sirtuin 1 (SIRT1) possesses NAD-dependent deacetylase activity that controls phosphorylation of IR and IRS in insulin independent-manner. It was found that SIRT-1 positively regulates insulin signaling via control of IRS-1 phosphorylation, deacetylation of IRS-2, repression of Ptpn1 expression and Akt activation in insulin-sensitive cells [102]. It was also found that the supplementation with 1,25(OH)_2_D_3_ improved metabolism of glucose via upregulation of the SIRT1/IRS1/GLUT4 signaling cascade and uptake of glucose in high glucose-treated C2C12 myotubes [103].

Peroxisome proliferator-activated receptor delta (PPAR-δ) is a transcription factor involved in the metabolism and mobilization of fatty acids in adipose tissue and skeletal muscle. It was shown that 1,25(OH)_2_D might activate PPAR-δ that in turn improved insulin sensitivity. 1,25(OH)_2_D_3_-mediated activation of PPAR-δ decreases FFAs-induced insulin resistance in skeletal muscle [96]. The action of vitamin D in reduction of insulin resistance in skeletal muscle is also associated with the regulation of intracellular Ca^2+^ level. Increased Ca^2+^ concentration enhances translocation of GLUT4 to the cell membrane in muscle cells and glucose uptake [104]. However, Alkharfy et al. have not found any changes in the expression of IRs and GLUT4 expression in muscle, adipose tissue, and liver of mice fed with low-fat diet or high-fat diet after supplementation of vitamin D [100].

Vitamin D deficiency is also related to increased levels of PTH associated with insulin resistance [105,106]. PTH may elevate the concentration of free intracellular Ca^2+^ in insulin-responsive tissues, including skeletal muscle and adipose tissue [107,108]. The results of the study focused on PTH treatment of osteoblast-like cell type have shown decreased insulin stimulated glucose transport [109]. Another study have indicated that PTH reduced glucose uptake stimulated via insulin in rat adipocytes [86]. It may suggest that PTH may evoke insulin resistance via decreasing the number of GLUT1 and GLUT4 in cell membranes thereby decreasing uptake of glucose [110]. Thus, PTH promotes insulin resistance via reduction of glucose uptake in adipose tissue, liver, and muscle [49].

It should be emphasized that vitamin D may also affect insulin resistance indirectly via renin–angiotensin–aldosterone system (RAAS). RAAS is known for its inhibitory effects on insulin action in peripheral tissues, and regulation of cellular Ca^2+^ level in skeletal muscle cells. This regulation may promote transport of glucose via membrane as a result of GLUT4 recruitment [104,111,112]. Moreover, angiotensin II induces generation of ROS via NADPH activating NF-κB, which in turn triggers insulin resistance in skeletal muscle [111]. The expression of renin and production of angiotensin II have been elevated in VDR-null mice and 1,25(OH)_2_D_3_ administration inhibited biosynthesis of renin [113,114,115]. Thus, vitamin D may improve insulin sensitivity via inhibition of RAAS [116]. Interestingly, vitamin D insufficiency is also connected with elevated infiltration of fat in skeletal muscle that appears independently of body mass and seems to contribute to decreased action of insulin [117].

Vitamin D was found to exert an effect on hepatic lipogenesis and gluconeogenesis. This action may be mediated via various vitamin D-regulated pathways including AMP-activated protein kinase (AMPK)–calmodulin and Akt/Notch signaling. AMPK is an enzyme regulating metabolism that is activated by phosphorylation through either the calcium/calmodulin protein kinase beta (CaMKKβ) or serine/threonine kinase 11 pathways [118]. Hepatic AMPK activation is characterized by the anti-diabetic actions including attenuation of gluconeogenesis and lipogenesis and the promotion of glycolysis and lipid oxidation [119]. Moreover, the activation of hepatic AMPK inhibits activity of Foxo1 [120] resulting in the reduction of hepatic ER stress and alleviation of insulin resistance and hepatic steatosis [121,122]. Leung et al. have found that high doses of 1,25(OH)_2_D_3_ were able to ameliorate the abnormal hepatic glucose and lipid metabolism in models of insulin resistance without any symptoms of toxicity. This was confirmed by the Lin et al. who showed that increased levels of cytosolic 1,25(OH)_2_D_3_ in HepG2 cells leads to the activation of Ca^2+^/CaMKKβ/AMPK pathways which, in turn confirm regulatory effects of calcitriol on glucose and lipids [123].

It was also demonstrated that elevated formation of ROS is an important activator of insulin resistance [124,125]. Oxidative stress coexisting with diabetes may be a result of increased level of FFAs acting on the mitochondria to increase ROS production (such as hydrogen peroxide, superoxide, hydroxyl radical ions) [126,127]. It was found by Inoguchi et al. that high glucose level and FFAs may stimulate ROS production via PKC-dependent activation of NADPH oxidase [128]. Interestingly, it was also demonstrated that vitamin D deficiency is related to decline in mitochondrial respiration deriving from the reduction of the nuclear mRNA molecules and proteins involved in this process [129,130]. Reduced respiration causes decline in mitochondrial bioenergetics following alteration in mitochondrial oxidative phosphorylation, decreasing ATP formation and increasing ROS production [50]. Especially, decreased expression of complex 1 of the electron transport chain leads to the reduction of ATP production and increase of ROS production. Elevated level of ROS decreases the activity of the insulin signaling pathways via serine/threonine phosphorylation of IRS, reduction of GLUT4 gene transcription, disturbances of insulin signaling redistribution in cell and alterations of mitochondrial activity [131]. A potential role of vitamin D in maintaining normal function of mitochondria may explain the link between diabetes and vitamin D deficiency.

It was also proposed that vitamin D maintains the control of cellular bioenergetics in mitochondria [132] and is able to regulate mitochondria function. Mechanism of vitamin D action via VDR in the nucleus leads to increasing expression of numerous components involved in mitochondrial function, including mitochondrial respiration [129,130]. Moreover, VDR is able to enter mitochondrion via permeability transition pores [133] and directly regulates its functions, but this mechanism is still not fully elucidated [134]. Suppressive effect of 1,25(OH)_2_D_3_/VDR signaling on differentiation of brown adipose cells and mitochondrial respiration was also observed [135]. VDR also plays a key role in protecting cells from excessive ROS production and excessive respiration that contributes to cell damage [136]. Vitamin D is involved in mitochondrial respiration balance via maintaining the activity of mitochondrial respiratory chain [137] and regulation of uncoupling protein 1 (UCP1) expression. UCP1 is localized on the inner membrane of mitochondria and responsible for control of thermogenesis [8].

Interestingly, vitamin D might also reduce formation of ROS in adipocytes [138] via controlling of cellular antioxidants expression [139]. It was shown that vitamin D together with Nrf2 and Klotho may regulate expression of numerous antioxidants. Vitamin D was documented to downregulate NADPH oxidase (NO_X_) that produces ROS [140] while upregulates superoxide dismutase (SOD) that is responsible for converting superoxide into hydrogen peroxide [141]. Moreover, vitamin D elevates the production of glutathione (GSH)—the major redox buffer via upregulating glutamate cysteine ligase, glucose-6-phosphate dehydrogenase (G6PD), and glutathione reductase [142,143,144].

### 4.3. Vitamin D Alters Epigenetic Modifications Evoked by Upregulation of DNA Demethylases Genes

**Figure 4:**
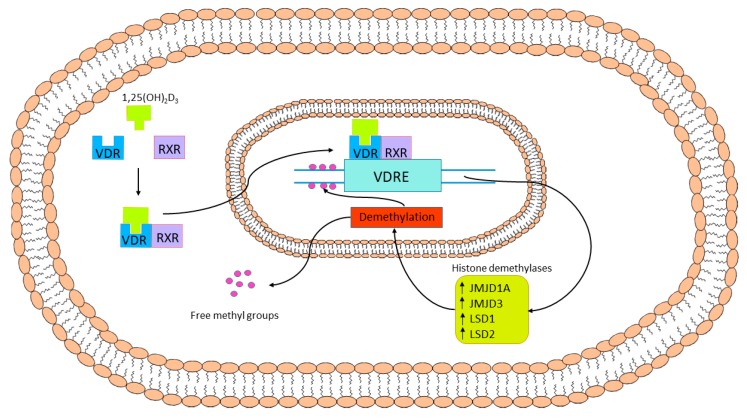
Genomic mechanism of vitamin D action involved in the regulation of DNA demethylases genes expression. The 1,25(OH)_2_D_3_ binds to VDR, which in turn heterodimerizes with RXR. The formed 1,25(OH)_2_D_3_-VDR-RXR complex translocates to the nucleus where it binds to VDRE. As a result, the expression of vitamin D-dependent DNA demethylases, i.e., LSD1, LSD2, JMJD1A, and JMJD3, is upregulated. These enzymes prevent hypermethylation of promotor regions of numerous genes.

It was found that in obese individuals, DNA methylation is increased and determined as one of the risk factors for development of diabetes [145]. Scavenger Receptor Class A Member 3 (SCARA3) and Peroxiredoxin-2 (*PRDX2*) are hypermethylation-inactivated genes, which result in increase of ROS level. *SCARA3* and *PRDX2* genes encode proteins reducing level of ROS [146]. It should be emphasized that vitamin D was found to maintain the expression of DNA demethylases genes. This effect of vitamin D is exerted via its genomic mechanism of action. In this way vitamin D regulates the expression of vitamin D-dependent DNA demethylases, i.e., lysine-specific demethylase 1 and 2 (LSD1 and LSD2) and Jumonji domain-containing protein 1A and 3 (JMJD1A and JMJD3). These enzymes prevent hypermethylation of promotor regions of numerous genes [50,147]. Genomic mechanism of vitamin D action involved in regulation of DNA demethylases genes expression is presented in Figure 4.

### 4.4. Is Vitamin D the Stimulator or Inhibitor of Adipogenesis?

Adipose tissue is an important endocrine, metabolic organ. It plays a significant role in glucose homeostasis and energy balance [148]. Adipose tissue is recognized as: white adipose tissue (WAT) and brown adipose tissue (BAT). WAT is localized in visceral and subcutaneous depots. In turn, BAT is unique adipose tissue type occurring in mammals and plays a role in regulation of body temperature. It was thought that BAT is present only during neonatal stage in human. However, it was recently identified that BAT also occurs in adult life of human [149].

Adipogenesis is a process of sequential stages of differentiation leading to the formation of mature adipocytes. Mature adipocyte is able to perform numerous functions such as secretion of adipokines, responding to insulin signaling, traffic of fatty acids across the membrane and synthesis of lipids [150]. Differentiation of preadipocytes to mature adipocytes involves intracellular signaling molecules, i.e., SMAD proteins [151], ribosomal protein S6 kinase 1 (S6K1) [152], and janus kinase-signal transducer and activator of transcription 3 (JAK-STST3) [153], affecting adipogenic transcription factors. Notably, adipocytes differentiation is regulated via numerous transcriptional factors such as sterol regulatory binding protein 1 (SREBP1), the master regulator od PPARγ and regulator CAAT/enhancer binding proteins (C/EBPβ, C/EBPα, C/EBPδ) [154,155]. The role of these transcriptional factors is the induction of expression of numerous genes involved in lipolysis, lipogenesis, and insulin sensitivity such as glucose transporter (GLUT4), lipoprotein lipase (LPL), fatty acid synthase (FASN), and fatty acid binding protein (FABP4) [156,157,158].

It was also demonstrated that vitamin D action is involved in the regulation of adipogenesis. VDR is expressed in adipocytes in early stages of adipogenesis and its level gradually decreases with the differentiation progress [159,160]. However, the results of the studies focused on the effect of 1,25(OH)_2_D_3_ on adipogenesis are inconclusive. Opposite to the inhibitory effect of vitamin D on adipogenesis in 3T3-L1 mouse preadipocytes cell line [160] and suppression of brown adipocyte differentiation [8], vitamin D was found to promote adipogenesis in primary mouse and human preadipocytes [161]. It was shown that a high level of 1α,25(OH)_2_D_3_ may inhibit early stages of adipogenesis in 3T3-L1 cells [159,160]. Calcitriol suppresses adipogenesis via exerting an effect on multiple targets inhibiting the expression of PPARγ and C/EBPα, antagonizing activity of PPARγ sequestrating RXR and decreasing mRNA and nuclear protein expression of C/EBPβ [159]. The 1,25(OH)_2_D_3_ induces expression of eight twenty-one (ETO)–C/EBPβ corepressor that, in turn, suppresses action of C/EBPβ transcriptional action required for adipogenesis [160]. Numerous signaling molecules, including members of the WNT family are secreted at the stage of preadipocyte differentiation [162]. Normally, the WNT/β-catenin pathway is downregulated during adipogenesis and maintain the preadipocytes in undifferentiated state [162]. Vitamin D contributes to the inhibition of adipocyte differentiation via MAPK [163] and Wnt/β-catenin signaling pathways [8,164]. It was demonstrated that 1α,25(OH)_2_D_3_ maintained the nuclear β-catenin and WNT10B expression, thereby inhibiting PPARγ and showing anti-adipogenic effect in 3T3-L1 preadipocytes [164]. Calcitriol was also reported to decrease the level of secreted fizzled-related protein 2 (SFRP2) expression via VDR-mediated WNT signaling leading to the inhibition of differentiation of mouse bone marrow stromal cells (BMSCs) [165]. Moreover, 1α,25(OH)_2_D_3_ was presented to inhibit both the mRNA expression and phosphorylation of extracellular regulated kinase (ERK), thus triggering the inhibition of adipocyte differentiation [163]. In addition, calcitriol was found to elevate the expression of adipogenic markers LPL and FABP4 promoting differentiation of human subcutaneous preadipocytes [161].

Interestingly, the exposure of porcine mesenchymal stem cells (MSCs) to vitamin D induced both proliferation and differentiation by increasing mRNA expression of adipocyte-binding protein 2 (AP2) LPL, and PPARγ [166]. It has been also shown that mesenchymal cells undergo differentiation towards adipocytes with accompanied increased expression of FABP4, FASN, and PPARγ as well as enhanced accumulation of lipids as a results of 1,25(OH)_2_D_3_ exposure [167].

To conclude, vitamin D exerts an effect on the expression of genes that play a key role in adipogenesis. However, taking into consideration inconclusive results, the vitamin D action on adipogenesis requires further studies.

### 4.5. The Role of Vitamin D in Adipocyte Apoptosis

**Figure 5:**
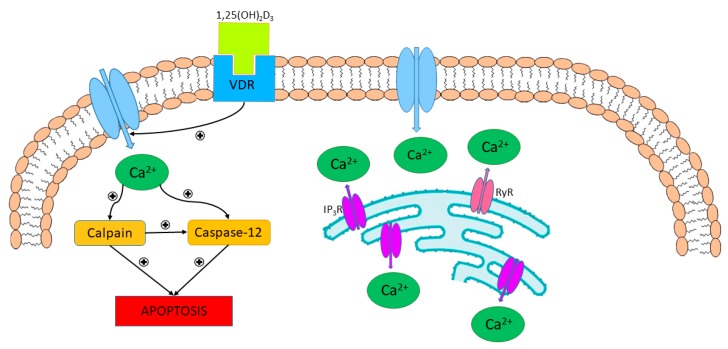
The 1,25(OH)_2_D_3_-mediated induction of adipocyte apoptosis. The 1,25(OH)_2_D_3_ stimulates both voltage-insensitive and voltage-dependent Ca^2+^ influx in mature adipocytes leading to the release of Ca^2+^ from ER stores via RyR and InsP_3_R. Increased intracellular Ca^2+^ level activates apoptosis via the Ca^2+^-dependent protease calpain contributing to the activation of the Ca^2+^/calpain-dependent caspase-12. Modified according to Abbas et al. [8]. 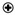
—activation.

Sun et al. have shown that calcitriol inhibited apoptosis and stimulated the expression of genes that favored proliferation in human subcutaneous adipocytes [168]. Interestingly, high doses of calcitriol stimulated, whereas its low doses suppressed apoptosis in differentiated 3T3-L1 cells [169]. It is suggested that low doses of vitamin D_3_ inhibits apoptosis via increasing mitochondrial potential and ATP yield as well as suppressing UCP2 [170]. Bioactive form of vitamin D_3_ stimulates both voltage-insensitive and voltage-dependent Ca^2+^ influx in mature adipocytes. It leads to the release of Ca^2+^ from ER stores via RyR and InsP_3_R. [171]. Elevated intracellular Ca^2+^ level activates apoptosis via the Ca^2+^-dependent protease calpain leading to the activation of the Ca^2+^/calpain-dependent caspase-12 [172]. The 1,25(OH)_2_D_3_-mediated induction of adipocyte apoptosis is presented in Figure 5.

### 4.6. Vitamin D Reduces Sub-Inflammation Coexisting with Insulin Resistance

The major site of vitamin D storage in an organism is adipose tissue [8]. This tissue expresses both VDR [173] and enzymes participating in metabolism of vitamin D [174]. Recent evidence indicates that vitamin D interacts with inflammatory involved adaptor molecules, membrane receptor, nuclear coregulatory proteins, and phosphatases in adipose tissue. Thus, vitamin D is involved in cell signaling and the control of gene expression [175].

The association between obesity and vitamin D deficiency is intensively investigated. Obesity is characterized by hypertrophic enlargement of adipose tissue resulting in improper blood flow, which in turn results in macrophages infiltration, hypoxia, and inflammation [176]. A special feature of the hypertrophied adipocytes is increased release of pro-inflammatory cytokines such as *IL-8*, *IL-6*, *TNF*-α, MCP1, and resistin, as well as decreased secretion of adiponectin [177,178,179]. Thus, one of consequences of obesity is altered secretion of adipokines. It is believed that dysregulation of numerous pathways in hypertrophic adipose tissue leads to the onset of insulin resistance [180]. It is known that vitamin D reduces insulin resistance-related inflammation [181]. It is suggested that this effect is connected with modulation of adipokines secretion, such as adiponectin and leptin, by vitamin D [182,183,184].

It was proposed that adiponectin, an anti-inflammatory and insulin-sensitizing hormone, is a biomarker of insulin resistance [185,186]. Its biological activity depends on its serum concentration, the type of isoforms, and receptor subtype specific to tissue. The negative correlation between circulating adiponectin and body mass index (BMI) has been demonstrated. The downregulation of adiponectin, especially the HMW isoform has been observed in obese children with vitamin D deficiency [8,184]. Significant increase in adiponectin level was found in T2DM patients supplemented with vitamin D-fortified food [187]. It has been also observed that vitamin D_3_ treatment increased expression of adiponectin and disulfide bond-A oxidoreductase-like protein (DsbA-L). DsbA-L is a protein regulating multimerization of adiponectin [184]. Interestingly, no effect of 1α,25(OH)_2_D_3_ on adiponectin expression in human adipocyte culture was found [188].

Leptin released by adipose tissue acts on the hypothalamus resulting in the reduction of appetite [189]. This hormone regulates metabolism of lipid via the stimulation of lipolysis and inhibition of lipogenesis [190,191]. Increased level of leptin is the stimulus to the brain that inhibits appetite and increases energy expenditure [189]. Leptin synthesis is stimulated by glucocorticosteroids, insulin, estrogens, and TNF-α, whereas it is inhibited by growth hormones and FFAs [192]. Positive correlation between leptin level and body fat mass has been observed [193]. Vitamin D plays an important role, not only in the regulation of adipokines secretion, but also in the control of energy homeostasis via regulation of leptin formation. It was demonstrated that vitamin D inhibited leptin secretion by adipose tissue [194]. Interestingly, CYP27B1 knockout mice were hypoleptinemic and consumed significantly more food than their wildtype counterparts. VDR knockout (VDRKO) mice had been characterized by hypoleptinemia, lean phenotype, and hyperphagia related to low level of leptin in the serum [195]. The serum level of leptin is determined by adipose tissue mass. It was not fully known whether hypoleptinemia is an effect of body fat content or is a direct effect of vitamin D/VDR system on leptin expression in VDRKO mice. Bioactive form of vitamin D_3_ directly stimulated expression and secretion of leptin in wild-type mouse adipose tissue cultures, but not from VDR-null mice adipose tissue cultures. It has been also demonstrated that calcitriol downregulates leptin by at least 84% in mouse 3T3-L1 adipocytes [196].

Interestingly, it was also observed that leptin inhibited renal activation of vitamin D_3_ to 1α,25(OH)_2_D_3_ indirectly via the stimulation of osteoblast or osteocyte FGF-23 production, or both [197]. As mentioned above, FGF-23 suppresses the synthesis of 1α,25(OH)_2_D_3_ via inhibiting renal CYP27B1. Impairment of WAT development and secretion of leptin occurs in Vdr^−/−^ and CYP27B1^−/−^ mice [198].

Bioactive form of vitamin D is known from its immunomodulatory function [199]. The results of several in vitro studies have shown that calcitriol suppresses chronic inflammation in human adipocytes and mouse 3T3-LI cell line [200,201]. The newest evidence has demonstrated that vitamin D-mediated reduced secretion of pro-inflammatory cytokines is responsible for diminishment of adipose tissue inflammation [202]. WAT accumulates macrophages that release pro-inflammatory cytokines, including TNF-α that play a crucial role in the development of insulin resistance [8]. Calcitriol suppresses secretion of MCP-1 induced by TNF-α, but inhibits adiponectin secretion in differentiated adipocytes from subcutaneous WAT [203]. Moreover, 1,25(OH)_2_D_3_ decreased IL-1β-induced expression of pro-inflammatory genes such as *IL-6*, *IL-8* and *MCP-1*. However, the results of in vitro studies are not in accordance with in vivo results. It was also shown that oral supplementation with 700 IU of vitamin D per day for 26 weeks had effect on the level of inflammation markers in obese patients [204]. In turn, in mice model of high-fat diet, supplementation with calcitriol reduced *IL-6* level in adipose tissue [200].

**Figure 6:**
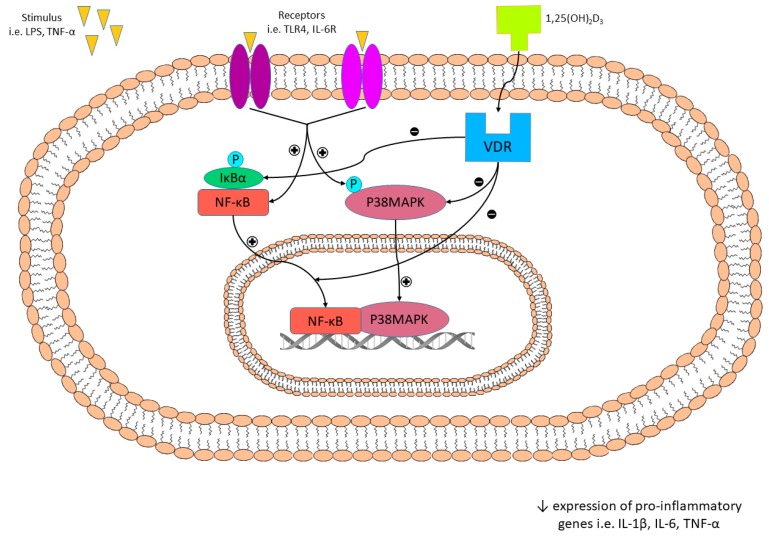
The inhibitory effect of 1,25(OH)_2_D_3_ on inflammation. LPS- or TNF-α-stimulated receptors i.e., TLR, *IL-6*R activates P38MAPK- or NF-κB-dependent transcription of pro-inflammatory genes such as *IL-1β*, *IL-6*, *TNF-α*. The 1,25(OH)_2_D_3_ inhibits inflammation via suppression of IκBα phosphorylation and subsequent translocation of P38MAPK or NF-κB into the nucleus, leading to decreased expression of pro-inflammatory genes. 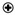
—activation, 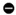
—inhibition.

It should be also emphasized that NF-κB is an essential component of inflammatory pathways in adipose tissue. The activation of NF-κB and translocation of p65 subunit to the nucleus is related to IκBα degradation [205]. It has been shown that calcitriol suppressed release of LPS-stimulated *IL-6* in differentiated MSC and human mature adipocytes [206]. An inhibitory effect of vitamin D on inflammatory markers in human and mouse adipocytes via NF-κB and p38 MAP kinase inflammatory pathway was also demonstrated [201,207,208]. LPS- or *TNF-α*-stimulated receptors such as *TLR*, *IL-6R* activates p38MAPK- or NF-κB-dependent transcription of pro-inflammatory genes including *IL-1β*, *IL-6*, *TNF-α*. The 1,25(OH)_2_D_3_ suppresses inflammation via inhibition of IκBα phosphorylation and subsequent translocation of P38MAPK or NF-κB into the nucleus [202]. The inhibitory effect of 1,25(OH)_2_D_3_ on inflammation is presented in Figure 6.

Cytokines may also stimulate both the IKK-β/NF-κB and Jun N-terminal kinase 1 (JNK1) pathways. In turn, these activated kinases may phosphorylate IRS-1, leading to the reduction of insulin signaling [50,209]. Thereby, excessive secretion of pro-inflammatory cytokines results in dysregulation of lipid and glucose metabolism [210]. Increasing evidence suggests that vitamin D reduces monocyte chemotaxis and secretion of cytokines and chemokines playing a key role in inflammation [8].

Recent evidence indicates that vitamin D exerts not only anti-inflammatory effect, but also it modulates immune system function [211]. Undoubtedly, vitamin D reduces adipose tissue inflammation acting on leukocyte infiltration and maturation of adipocytes [212,213]. Vitamin D is able to affect the action on both innate and adaptive immune system [214]. Its effect on dendritic cells comprises elevated production of anti-inflammatory *IL-10* and decreased release of pro-inflammatory cytokines such as *TNF-α*, *IL-12*, and *IFN-γ*. Moreover, dendritic cells acquire tolerogenic properties and peculiar immunoregulatory role as a result of vitamin D exposure [215]. In monocytes, vitamin D decreases the expression and production of pro-inflammatory cytokines such as *IL-1β*, *TNF-α*, *IL-6*, and *IL-8* [216,217]. Interestingly, in lymphocytes, vitamin D participates in switch from more inflammatory response of T-helper 1 (Th1)/Th17 to profile of less inflammatory Th2/Treg [218]. Since adipose tissue contains a large amount of immune cells, it plays a key role in the maintenance of immune homeostasis [219]. The activity of T lymphocyte is modulated in obesity [220]. Regulatory T cells (Treg) are the subtypes of T lymphocytes that are significantly reduced in VAT of obese mice [221]. Recently, two crucial mechanisms related to insulin resistance were clarified, dependent and not dependent on obesity. Obesity-related insulin resistance is attributed to macrophages-driven inflammation [222]. Obesity-independent, age-related insulin resistance is regulated via adipose-resident regulatory T cells (aTregs) [223]. Vitamin D_3_ decreases inflammation due to the ability to enhance the suppressive activity of Tregs [221]. Unfortunately, the effect of vitamin D on aTregs is still not fully elucidated [224].

To conclude, vitamin D is a potential negative modulator of pro-inflammatory cytokines release [208,225], reducing *IL-6*, *TNF-α*, and C-reactive protein [226], and it exerts a significant effect on the immune system and adipose tissue [208,227]. Calcitriol also strongly suppresses the activation of MAPK and NF-κB signaling pathways, preventing transcription of pro-inflammatory factors genes. Thus, the bioactive form of vitamin D significantly reduces inflammation in adipose tissue.

## 5. Conclusions

In the face of an epidemic of diseases associated with insulin resistance, the beneficial effects of vitamin D has attracted an attention of many researchers, clinicians, and health specialists. Recent findings suggested that the molecular background of insulin resistance formation is connected with vitamin D deficiency. Both genomic and non-genomic molecular action of vitamin D are involved in the maintenance of insulin sensitivity. These favorable effects are not only related directly to insulin signaling, but also indirectly with reduction of oxidative stress, sub-inflammation and epigenetic regulation of gene expression as well as RAAS. However, few studies have not confirmed these desired effects of vitamin D on insulin-sensitive tissues. Taken together, the result of basic and clinical studies revealed that vitamin D deficiency is a crucial factor that may accelerate insulin resistance formation. Deeper understanding of vitamin D molecular involvement in processes related to insulin signaling may result in new therapeutic strategies preventing from development of insulin-resistance-associated disorders. 

## Figures and Tables

**Table 1:** Classification of diagnostic vitamin D cut-offs based on 25(OH)D (calcidiol) concentration [24].

Concentration of Calcidiol (nmol/L)	Concentration of Calcidiol (ng/mL)	Classification
<50	<20	Deficiency
50–80	20–32	Insufficiency
135–225	54–90	Normal (in sunny countries)
>250	>100	Excess
>325	>150	Intoxication

**Table 2:** Observational studies on the association between vitamin D levels and metabolic parameters involved in insulin resistance.

Study Design	Target Population	Studied Parameters	Main Effect	Reference
Observational, cohort, cross-sectional	358 men completed the study	25(OH)D, hs-CRP, HOMA-IR, FPI, FPG, TG, DBP, SBP, waist circumference, BMI	Positive	[52]
Observational, cohort, cross sectional	4116 non-diabetic adults	25(OH)D, FPI, FPG, BMI, DBP, SBP, weight, waist circumference, age, sex	Positive	[53]
Cohort, cross sectional	1074 man with and without diabetes	25(OH)D, HbA1c, lipid profile	Positive	[54]
Cohort, cross sectional	157 pre-diabetes patients	25(OH)D, FPI, FPG, QUICK, HOMA2-IR, HOMA-β	Positive	[55]
Cohort, cross sectional	5867 adolescents	25(OH)D, SBP, CRP, lipid profile, waist circumference, HOMA-IR	Positive	[56]
Observational, cohort, cross sectional	3691 patients with T2DM	25(OH)D, HOMA-IR, IGI/IR, ISSI-2, PTH, BMI	Positive	[57]
Observational, cohort, cross sectional	712 patients with risk factor of T2DM	25(OH)D, HOMA-IRMatsuda insulin sensitivity index, IGI/IR, ISSI-2	Positive	[58]
Observational	39 patients with no known history of diabetes	25(OH)D, PTH, TC, HDL, LDL, BMI, TG	Positive	[59]
cohort, cross sectional	126 healthy patients with glucose tolerance	25(OH)D, first- and second-phase insulin responses (1st IR and 2nd IR), ISSI	Positive	[60]

Abbreviation: DBP, diastolic blood pressure; SBP, systolic blood pressure; BMI, body mass index; FPI, fasting plasma insulin; FPG, fasting plasma glucose; hs-CRP, high sensitive C-Reactive Protein; HDL, high-density lipoprotein, TG, triglycerides; HOMA-IR, Homeostatic Model Assessment for Insulin Resistance; BMI, body mass index; HbA1c, glycated hemoglobin; QUICK, Quantitative Insulin Sensitivity Check Index; HOMA-β, Homeostatic Model Assessment of β-cells Function; IGI, Insulinogenic index; ISSI-2, Insulin secretion sensitivity index-2; PTH, parathyroid hormone; LDL, low-density lipoprotein.

**Table 3:** The result of interventional clinical trials focused on the effect of vitamin D supplementation on metabolic parameters involved in insulin resistance.

Study Design	Target Population	Duration	Dosage	Studied Parameters	Main Effect	Reference
Paralleled, double-blinded, randomized, placebo-controlled clinical trial	50 patients with diabetic nephropathy and marginal serum vitamin D level	8 weeks	Intervention group received 50,000 IU/week of 1,25(OH)_2_D_3_ (*n* = 25), placebo group (*n* = 25) received an identical placebo	Lipid profiles (LDL, HDL, TG and TC), oxidative/anti-oxidative markers (TAC, CAT, SOD, GPx and MDA)	Positive/Neutral	[5]
Parallel group, randomized, placebo-controlled trial	60 patients with T2DM and hypovitaminosis D	6 months	60,000 IU of oral vitamin D every week for first six weeks and then once every 4 weeks till the end of the study; microcrystalline cellulose constitutes oral placebo	Vitamin D levels, HbA1c and vitamin D levels, FPG, PPPG, TC, LDL	Positive	[4]
Randomized, controlled trial	115 subjects with vitamin D deficiency	6 months	Intervention group received 30,000 IU of cholecalciferol/week	HOMA-IR, 25(OH)D, FBG, HbA1c, BMI, FBI, TC, LDL, HDL, PTH	Positive	[7]
Double-blind, randomized, controlled trial	130 men with 25(OH)D levels < 50 nmol/L and without diabetes	1 year, evaluation after 6 and 12 months	100,000 IU of vitamin D bimonthly or placebo	25(OH)D, FPG, hs-CRP, insulin, lipid profile, anthropometric measures	Positive	[61]
Double-blind, randomized, placebo-controlled trial	340 non-diabetic adults with increased risk of T2DM	4 months, evaluation between baseline and 4 months	100,000 IU of vitamin D_2_ vs. 100,000 IU of vitamin D_3_ vs. placebo	HbA1c, blood pressure, lipid and CRP as well as apolipoprotein levels, PWV, anthropometric measures	Neutral	[62]
Double-blind, randomized placebo-controlled trial	16 patients with T2DM and D hypovitaminosis	12 weeks	280 µg daily of vitamin D for 2 weeks, 140 µg daily of vitamin D for 10 weeks, placebo for 12 weeks	25(OH)D, lipid profile, C peptide, plasmatic calcium, inflammation markers, insulin sensitivity, insulin after IVGTT, insulin pulsatility, ABPM	Neutral	[63]
Placebo-controlled, randomized clinical trial	118 non-smoker subjects with T2DM and vitamin D insufficiency	8 weeks	1st group: 50,000 U/week vitamin D + calcium placebo; 2nd group:1000 mg/day calcium + vitamin D placebo; 3rd group: 50,000 U/week vitamin D + 1000 mg/day calcium 4th group: vitamin D placebo + calcium placebo	Serum insulin, HbA1c, HOMA-IR, LDL, total/HDL-cholesterol, QUICK, HOMA-β, HDL	Positive	[3]
Double-blinded, randomized control study	109 prediabetes subjects with vitamin D deficiency	weekly vitamin D or placebo	doses based on body weight and baseline levels of 25(OH)D	25(OH)D, HbA1c, insulin secretion, insulin sensitivity, 2 h glucose, FPG	Neutral	[64]
Double-blind, randomized, placebo-controlled clinical trial	48 healthy pregnant women (at 25 weeks of gestation)	9 weeks	400 IU/day of cholecalciferol supplement or placebo	25(OH)D, insulin, hs-CRP, blood pressure, plasmatic calcium, lipid concentrations, FBG, biomarkers of oxidative stress	Positive	[65]
Double-blinded, randomized clinical trial	42 patients with diabetes	evaluation 3 months after injection	single intramuscular injection of 300,000 IU of vitamin D_3_ in intervention group	25(OH)D, HbA1c, HOMA, BMI, insulin, blood glucose, blood pressure, waist circumference	Neutral	[66]
Open label study	8 subjects with prediabetes and vitamin D deficiency	4 weeks	10,000 IU of vitamin D_3_ daily	Acute insulin response to glucose, IVGTT, insulin sensitivity, disposition index	Positive	[67]
Double-masked, placebo-controlled trial; 2-by-2 factorial-design	92 adults with T2DM risk	16 weeks	2000 IU of cholecalciferol once daily or 400 mg of calcium carbonate twice daily	25(OH)D, HbA1c, acute insulin response, glycaemia, plasmatic calcium, insulin sensitivity, disposition index after an IVGTT	Positive	[68]
Randomized, controlled trial	100 patients with T2DM	12 weeks	Plain yogurt drink containing 170 mg calcium and no vitamin D/250 mL or vitamin D_3_-fortified yogurt drink containing 170 mg calcium and 500 IU vitamin D/250 mL twice a day	Lipid profile, glycemic status, E-selectin, Endotelin-1, MMP-9, body FAT mass, anthropometric measures	Positive	[69]
Double-blinded, randomized controlled study	81 South Asian women	6 months	4000 IU of vitamin D_3_ or placebo daily	25(OH)D, lipid profile, CRP, C peptide, HOMA 1	Positive	[70]

Abbreviation: PPPG, post prandial plasma glucose; DBP, diastolic blood pressure; SBP, systolic blood pressure; BMI, body mass index; FPI, fasting plasma insulin; FPG, fasting plasma glucose; hs-CRP, high sensitive C-Reactive Protein; HDL, high-density lipoprotein, TG, triglycerides; TC, total cholesterol; HOMA-IR, Homeostatic Model Assessment for Insulin Resistance; BMI, body mass index; HbA1c, glycated hemoglobin; QUICK, Quantitative Insulin Sensitivity Check Index; HOMA-β, Homeostatic Model Assessment of β-cells Function; IGI, Insulinogenic index; ISSI-2, Insulin secretion sensitivity index-2; PTH, parathyroid hormone; LDL, low-density lipoprotein; TAC, Total Antioxidant Capacity; CAT, Catalase; SOS, Superoxide dismutase; Gpx, Glutathione peroxidase; MDA, Malondialdehyde; FBG, fasting blood glucose; FBI, fasting blood insulin; PWV, Pulse wave velocity; IVGTT, Intravenous glucose tolerance test; ABPM, Ambulatory blood pressure monitoring; MMP-9, Matrix Metalloproteinase-9.
